# The Role of AhR in Autoimmune Regulation and Its Potential as a Therapeutic Target against CD4 T Cell Mediated Inflammatory Disorder

**DOI:** 10.3390/ijms150610116

**Published:** 2014-06-05

**Authors:** Conghui Zhu, Qunhui Xie, Bin Zhao

**Affiliations:** State Key Laboratory of Environmental Chemistry and Ecotoxicology, Research Center for Eco-Environmental Sciences, Chinese Academy of Sciences, Beijing 100085, China; E-Mails: toxdean@126.com (C.Z.); qhxie@rcees.ac.cn (Q.X.)

**Keywords:** aryl hydrocarbon receptor, inflammatory autoimmune disease, AhR ligand, drug target

## Abstract

AhR has recently emerged as a critical physiological regulator of immune responses affecting both innate and adaptive systems. Since the AhR signaling pathway represents an important link between environmental stimulators and immune-mediated inflammatory disorder, it has become the object of great interest among researchers recently. The current review discusses new insights into the mechanisms of action of a select group of inflammatory autoimmune diseases and the ligand-activated AhR signaling pathway. Representative ligands of AhR, both exogenous and endogenous, are also reviewed relative to their potential use as tools for understanding the role of AhR and as potential therapeutics for the treatment of various inflammatory autoimmune diseases, with a focus on CD4 helper T cells, which play important roles both in self-immune tolerance and in inflammatory autoimmune diseases. Evidence indicating the potential use of these ligands in regulating inflammation in various diseases is highlighted, and potential mechanisms of action causing immune system effects mediated by AhR signaling are also discussed. The current review will contribute to a better understanding of the role of AhR and its signaling pathway in CD4 helper T cell mediated inflammatory disorder. Considering the established importance of AhR in immune regulation and its potential as a therapeutic target, we also think that both further investigation into the molecular mechanisms of immune regulation that are mediated by the ligand-specific AhR signaling pathway, and integrated research and development of new therapeutic drug candidates targeting the AhR signaling pathway should be pursued urgently.

## 1. Introduction

The aryl hydrocarbon receptor (AhR) was initially identified in early toxicology studies that observed an increase in monooxygenase activity after exposure polycyclic aromatic hydrocarbons, which was involved in the metabolism of such environmental chemicals [1]. The AhR gene has been revealed as highly conserved throughout evolution, and the fact that the AhR gene has been so highly conserved provides evidence of the fundamental importance of the AhR in biological systems [2]. Current evidence also supports that the AhR plays an important role within biological systems across several species. In the mouse, for instance, targeted disruption of the AhR results in incomplete development of immune organs and altered immune function [3,4]. Other biological functions of AhR were also proposed in recent years, for example, that the AhR has a role in the female reproductive system including ovarian function, establishment of an optimum environment for fertilization, nourishing the embryo and maintaining pregnancy, as well as in regulating reproductive lifespan and fertility [5]. More recently, increasing experimental and epidemiology studies have shown that the AhR plays an important role in immune functions, particularly in the realm of CD4 helper T cell mediated inflammation [2,6,7]. The expression of the AhR in a majority of immune cell types and the expression of xenobiotic- or dioxin-responsive elements (XREs/DREs) in the promoter region of many genes that regulate the immune response demonstrates the importance of this receptor in immunological processes [8]. However, interestingly, paradoxical conclusions could be found when we reviewed the previous studies. For example, results determining whether the AhR is essential for the development of a functional immune system or whether the activation or suppression of AhR leads to profound immunosuppression, were not consistent [2,6,9,10,11,12]. Such functional diversity might depend on development stage, the diversity of ligand type or structure, the cell or tissue types involved, the microenvironment of the study system, and also the dose level.

**Figure 1:**
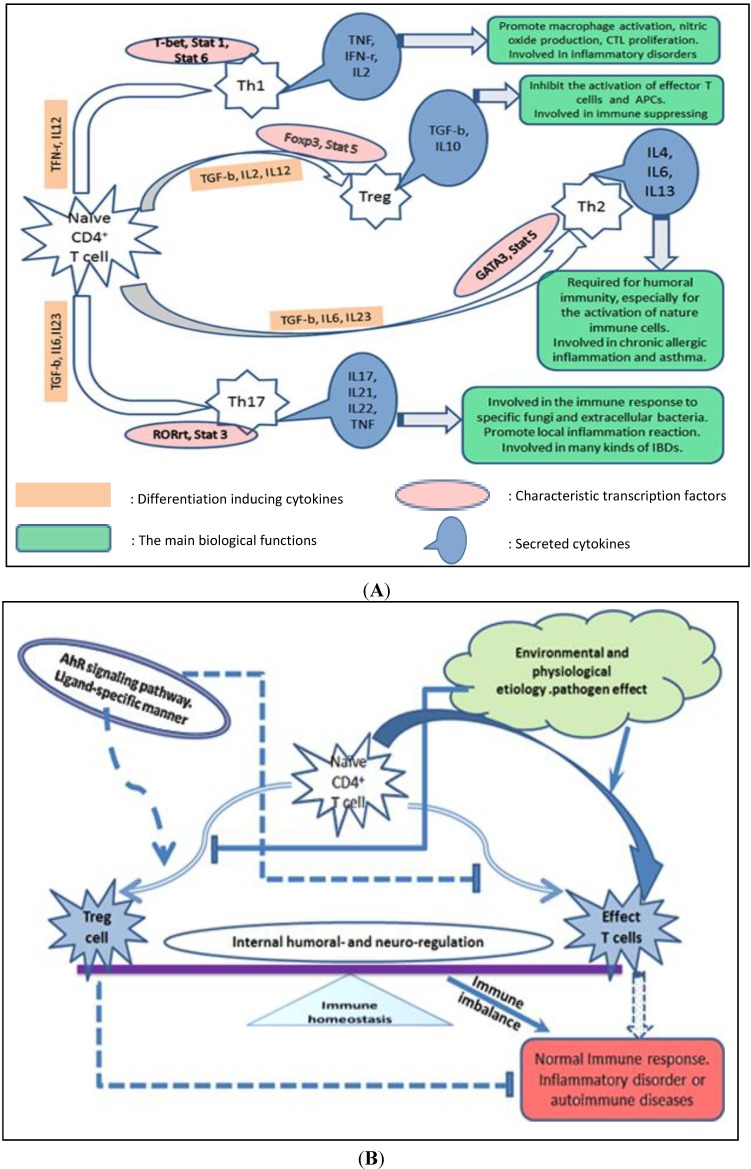
Diagram of CD4 T cell differentiation and immunomodulation by the AhR signaling pathway. (**A**) Summary of the major sets of CD4 T cells, including critical differentiation factors for fate determination, characteristic transcription factors, their unique products and some of their functions; (**B**) AhR signaling pathway involved in the differentiation of CD4 helper T cell subsets. Immune homeostasis in mammals is precisely controlled by internal humoral factors and neuro-regulation. When disturbed by environmental and physiological factors or by pathogens, homeostasis will be destroyed, and the resulting immune imbalance promotes inflammatory autoimmune diseases. The AhR signaling pathway can promote Treg cell differentiation, decrease effector T cells, especially the Th17 cell subset, directly or indirectly and in a ligand-specific manner.

As one of the main subgroups of the adaptive immune system, CD4 T cells play an integral role in adaptive immune responses. Activation of CD4 helper T cell requires recognition of the antigen in the context of MHC-II molecules (expressed on so-called professional APC such as Macrophage and DC), and then the activated effector T helper cells produce functional cytokines. Following activation, naive CD4 T cells differentiate into one of several lineages of T helper cells (Th1, Th2, Th17, or Treg), depending primarily on the stimulation factors, the strength of the TCR signal, and the cytokines present in the surrounding extracellular environment. Differentiation of each T cell subset is associated with the expression of specific transcription factors followed by secretion of a defined array of cytokines that mediate response to the antigen. Some of the major subsets of CD4 T cells, including their specific products (cytokines), the characteristic transcription factors and cytokines critical for their fate determination and some of their functions are summarized Figure 1A. Failure to activate an appropriate T cell response can lead to chronic infection, while exaggerated T cell responses can cause excessive tissue damage and are associated with inflammatory and autoimmune diseases. Th1 and Th17 are the two major functional helper T cell subsets that play important roles in the process of pro-inflammatory and inflammatory autoimmune disease [13,14,15].

Thus, a protective immune response is a trade-off between sterilizing immunity and its negative regulation. The actions of T helper cells can be balanced by CD4^+^CD25^+^ regulatory T (Treg) cells. Treg cell-mediated suppression of inflammation associated with autoimmunity is one of the most important regulatory mechanisms [16,17]. Besides the environmental factors that are believed to contribute to the increased prevalence of autoimmune diseases, breakdown of self-tolerance will also result in the development of inflammatory autoimmune diseases. Although immunological tolerance can be achieved by different mechanisms at different stages, both natural and induced CD4 regulatory T cells have been implicated in the regulation of autoimmune response [18]. The primary function of regulatory T cells involves suppression of successful immune responses and control of self *versus* non-self recognition. Failure of the latter results in autoimmune destruction of host cells and tissue. Taking advantage of the immunosuppressive potential of regulatory T cells is an important area for progress in the fields of autoimmune disease and organ transplantation.

Previous studies have demonstrated that activation of AhR by specific ligands regulates the generation of regulatory T cells, modulates the Th1 and Th2 balance [19], and increases the proportion of Th17 cells and their production of cytokines to promote the development of autoimmune diseases [3,6]. But notably, the immunomodulation effect of AhR might depend upon the interaction among AhR signaling pathway and other transcription factor signaling pathways involved in the differentiation or cytokines production of CD4 T helper cells subsets, and also such immunomodulation effects might occur in a ligand-specific fashion [6,20] (Figure 1B). AhR ligands can vary greatly in their chemical structure and binding affinities. Those AhR ligands which possess a greater affinity for the receptor are often deemed more toxic; however, researchers have been searching for additional, less toxic AhR ligands that possess immunomodulatory and anti-cancer properties [10,21].

The aim of the review is to discuss how the AhR signaling pathway is involved in the process of autoimmune disease and immune regulatory effects on inflammation mediated by CD4 helper T cell subsets. We also address the immunomodulatory roles of different AhR ligands in common inflammatory autoimmune diseases. In addition, the evidence-based potential of AhR as a therapeutic target against CD4 T cells mediating inflammatory disorders will be highlighted.

## 2. AhR Signaling Pathway and the Potential Molecular Mechanisms of Action Mediated by AhR Biological Signaling

The aryl hydrocarbon receptor (AhR) is a ligand dependent transcription factor that mediates many of the toxic or other biological effects of persistent organic pollutants such as halogenated aromatics (HAs) and polycyclic aromatic hydrocarbon (PAHs) related chemicals [22]. The best studied and highest affinity ligand for the AhR is 2,3,7,8-tetrachlodibenzo-*p*-dioxin (TCDD), which is formed as an unintentional by-product of incomplete combustion and released into the environment during the combustion of fossil fuels and wood and also during the incineration of municipal and industrial wastes. Many of the discoveries concerning the AhR and its properties were greatly enhanced by TCDD-related research.

The basic structure of the AhR, an orphan receptor, consists of an *N*-terminal basic helix loop helix domain, a *C*-terminal variable domain, and a central PER-ARNT-SIM (PAS) domain. It is important to note that the three dimensional structure of the AhR ligand binding domain has not been elucidated, and the mechanism underlying receptor conformational changes following binding remains unknown [23]. The inactive form of AhR normally exists in a multi-protein complex with specific chaperone proteins. Upon binding an exogenous or endogenous ligand, the cytoplasmic AhR translocates to the nucleus, where it binds to another protein known as the AhR nuclear translocator (ARNT). The ligand-activated AHR-ARNT complex is capable of binding to specific sequences of DNA (5'-TNGCGTG-3'), known as XREs or DREs. Once bound, the complex recruits appropriate transcriptional elements to increase or decrease target gene transcription. Most ligands are thought to signal through AhR by this mechanism [12].

The importance of the AhR signaling pathway in the process of both immune system development and immune modulation has been increasingly investigated since Funatake *et al.* first described the importance of AhR signaling in the generation of Tregs *in vivo* [24]. The potential molecular mechanisms of action of AhR involved in these processes have been investigated recently. Based on current information from the literature, we summarize five pathways involved in immune modulation of AhR.

### 2.1. Direct Effects of AhR on the Target Genes or the Transcription Factors Involved in Regulating Target Gene Expression.

The expression of AhR in a majority of immune cell types and the expression of multiple xenobiotic- or dioxin-responsive elements (XREs/DREs) in the promoter regions of many genes that regulate the immune response demonstrate the importance of this receptor in immunological processes [8]. AhR binding sites have also been identified in the promoter regions of several genes important in cell growth and differentiation, including genes for cell cycle control, growth factors, and growth factor receptors, which suggests that AhR activation may influence several basic homeostatic control mechanisms in the organism [25]. Previous studies have shown that AhR could be induced under Th17 polarizing conditions and involved in Th17 development. AhR participated in Th17 differentiation through regulation of Stat1 activation. Stat1 then binds to the IL17 promoter and serves as a repressor of Th17 differentiation [26]. However, there is evidence that AhR controls of Treg and Th17 cell differentiation might work in a ligand-specific and cell type-specific fashion [6].

### 2.2. AhR Regulates Differentiation and Cytokine Production of Th Cell Subsets Indirectly through Its Interaction with Other Types of Immune Cells

Cells of the immune system communicate with one another by releasing and responding to chemical messengers called cytokines. These proteins are secreted by different immune cells and act on other cells to coordinate appropriate immune responses. The local cytokine environment, including a diverse assortment of interleukins, interferons, and growth factors present at the early stage of naive CD4 T cell activation, is a critical factor that determines the development and differentiation of Th cells subsets. Macrophages are important for innate immune responses, producing pro-inflammatory cytokines, and contribute to TLR-mediated tuning of adaptive immune responses. A previous study demonstrated that LPS-induced production of such pro-inflammatory cytokines as IL-6, TNF-α and IL-12 is augmented in AhR^−/−^ peritoneal macrophages compared with WT cells. The molecular mechanism involved the interaction of AhR with Stat1 on the IL-6 promoter, which suppresses LPS-induced activation of IL-6 expression by inhibiting the transcriptional activity of NF-κB [27], while at the same time, Th17 differentiation is positively regulated by IL6 or IL21 in combination with TGF-b [26]. Dendritic cells (DCs) and their associated inflammatory cytokines can induce naive T-cell activation, proliferation and differentiation. Another study showed that activation of, or presence of DCs would increase TCDD-mediated apoptosis in activated, but not un-activated, peripheral T cells. The potential mechanism was thought to be due to TCDD-induced AhR expression in activated T cells, that further activated Fasl/Fas cell apoptotic signals [28]. Activation of AhR by Kyn promotes the induction of indoleamine 2,3-dioxygenase (IDO) by binding the Ido promoter that contains putative DRE consensus sequences. A recent *in vitro* study showed that IDO generated by DCs leads to increased Kyn formation that directly promotes the generation of Treg in an AhR-activation dependent manner, and also found that the cytokine, TGF-b, contributes to the Kyn binding affinity, further promoting AhR activation [29]. Nguyen *et al.* found that AhR is required to induce IDO expression, an immunosuppressive enzyme that catabolizes tryptophan into kynurenine (Kyn) and other metabolites in DC [30]. Reduced Kyn and IL-10 production was found in observed in mature bone marrow-derived dendritic cells (BMDCs) from AhR-deficient (AhR^−/−^) mice when compared with that from AhR^+/+^ mice, after LPS or CpG stimulation *in vitro*. Furthermore, their coculture system demonstrated that AhR^−/−^ BMDC inhibited naive T-cell differentiation into regulatory T (Treg) cells while facilitating Th17 cell development, while addition of synthetic L-Kyn reversed the situation in the coculture system.

### 2.3. AhR Regulation of Immune Response through Interaction with the Endocrine System Specific Sex Hormone Signaling Pathway

Previous studies showed that AhR ligands could also activate other transcription factor signaling pathways, especially the estrogen receptor signaling pathway [31]. One previous study demonstrated that estrogens contribute to the female preponderance of autoimmune diseases such as systemic lupus erythematosus (SLE) [32]. Although xenoestrogens may have compound- and tissue-specific effects that require further elucidation in future work, it is possible that environmental xenoestrogens superimposed upon the endogenous pituitary-gonadal axis may affect the development of autoimmunity [32]. Three-Methylcholanthrene (3MC) is an AhR agonist, and it has been reported that 3MC not only induces estrogenic activity through AhR-estrogen receptor alpha (ER alpha) interactions, but also can activate ER alpha-dependent transactivation directly [33]. Several studies have demonstrated ER expression in thymocytes and thymic epithelial cells in both mice and humans, and also found that ER played an important role in mediating the immunomodulatory effect of xenoestrogens [34]. DSS-induced colitis was ameliorated by pretreatment with the potent AhR activator, TCDD, in mice. In addition, mice pretreated with TCDD showed increased prostaglandin E2 (PGE2) production in the colon, and the inhibition of PGE2 production by indomethacin abrogated the inhibitory effects of TCDD on DSS-induced colitis [35].

### 2.4. AhR Regulation of Immune Cell Differentiation through the Epigenetic Modulation of Target Genes

The more detailed molecular basis by which AhR signaling modulates Treg biology is beginning to be understood. DNA methylation in a gene promoter region is associated with loss of that gene’s expression, and the methylation of the Foxp3 non-intronic upstream enhancer region could repress Foxp3 expression [36]. AhR signaling can also directly shape Treg differentiation by dictating the state of Foxp3 promoter methylation. Increased methylation of CpG islands of Foxp3 and demethylation of IL-17 promoters are found in DSS-induced acute colitis in C57BL/6 mice, interestingly, the treatment with TCDD mediates partial demethylation of the *Foxp3* promoter and hence enhances Foxp3 expression, while it mediates methylation of IL-17 promoter and decreases the expression of IL-17, a pro-inflammatory cytokine secreted by Th17 cells [37].

### 2.5. AhR Effect on Immune Cells by Modulation of Ubiquitin Ligase Activity

In eukaryotes, the ubiquitin proteasome system is employed in the selective degradation of many proteins, the E3 ubiquitin ligase directs myriad eukaryotic proteins to a variety of fates and functions [38]. A recent study demonstrated that AhR is also a ligand-dependent E3 ubiquitin ligase, implying that AhR has dual functions in regulating protein levels, serving both as a transcription factor that promotes the expression of target proteins and as a ligand-dependent E3 ubiquitin ligase that regulates selective protein degradation [39]. Besides, in addition to the genomic actions, a non-genomic action of ligand-activated AhR through the assembly of a CUL4B-based ubiquitin ligase complex and promoting the degradation of ERα has been found, such finding will further contribute to the understanding of the potential mechanisms of action on the interaction of ER signaling and AhR pathway [40]. These findings suggest that another potential mechanism of action for AhR might underlie its effects on the immune system.

## 3. AhR Ligands and Their Functional Diversity in CD4 Helper T Cells Mediate Common Inflammatory Autoimmune Diseases

Initial skepticism regarding the AhR as a drug target was primarily due to the traditional extensive toxicology studies demonstrating that most of the AhR ligands were toxic HAs and PAHs. However new AhR ligands, both endogenous and synthetic, has been identified, including bilirubin, indigoids, FICZ, kynurenine and several classes of chemoprotective phytochemicals such as the flavonoid and polyphenolic compounds, tryptophan and its derivatives (indole-3-carbinol and DIM), quercetin, curcumin, resveratrol and related compounds. Such AhR ligands can vary greatly in their chemical structure and binding affinities and such compounds have been shown to be less toxic but still able to elicit AhR signaling pathway responses. The representative structures of functional classes of AhR ligands have been summarized in previous work [22,41]. In the following text, we will discuss the existing AhR agonists, antagonists and their functional diversity in the common inflammatory autoimmune diseases that are mainly caused by CD4 T cell imbalance.

Based on the characteristic structure of AhR mentioned above, the promiscuity of the AhR ligand binding pocket is implied by its accommodation of a diverse group of ligands [41]. Many ligands of AhR and their potential bioactivities, including both the exogenous and endogenous agonists and antagonists, have been discovered in the past few years, which further demonstrates the potential role for AhR-targeted therapy. Ligand-specific differences have been observed during the investigation of mouse models of inflammatory autoimmunity [6]. AhR ligands can be classified into three groups: the agonists, the antagonists and the selective AhR modulators or SARMs Representative structures of functional classes of AhR ligands can be found in a review [41].

### 3.1. Crohn’s Disease and Inflammatory Bowel Disease

Aberrant CD4 T cells or imbalance of CD4 T cell subsets contribute to the genesis and prognosis of inflammatory autoimmune diseases. Crohn’s disease, also known as Crohn syndrome and regional enteritis, is a type of inflammatory bowel disease (IBD) that may affect any part of the gastrointestinal tract from mouth to anus, causing a wide variety of symptoms, which results from a combination of genetic and environmental factors that trigger an inappropriate immune response to commensal gut bacteria [42]. Since Crohn’s disease affects millions of people worldwide, it is essential to investigate the underlying mechanisms of pathogenesis so that new and more effective therapeutics can be developed. Additionally, activation of AhR may modulate immune and inflammatory responses, so studying the effects of AhR activation on the pathogenesis of Crohn’s disease is warranted [43].

Type 1 T helper (Th1) cells produce interferon-gamma (IFN-r), interleukin (IL)-2, and tumour necrosis factor (TNF), which activate macrophages and are responsible for cell-mediated immunity and phagocyte-dependent protective responses, and also are required for host defense against microbial pathogens. Th1 cells are involved in the pathogenesis of Crohn’s disease, acute kidney allograft rejection, and unexplained recurrent abortions. A recent study showed that pretreatment with TCDD suppressed inflammation in the gut of the 2,4,6-trinitrobenzenesulfonic acid (TNBS)-induced murine model of colitis. These results also demonstrated that TCDD activated AhR decreased pro-inflammatory cytokines levels of interleukin 6, IL-12, interferon-gamma, and tumor necrosis factor-alpha and induced the differentiation of Foxp3 Treg cell. Together these decreased colonic inflammation in a murine model of colitis in part by generating regulatory immune cells [44].

The pathogenesis of inflammatory bowel disease (IBD) is believed to involve an altered balance between effector and regulatory T cells. Intestine tissue from patients with IBD expressed significantly less AhR than controls [45], Experimental animal models of colitis show that attenuation of AhR receptor expression resulted in a protective effect during DSS-induced colitis, while the absence of AhR exacerbated the disease. Abnormal AhR pathway activation in the intestinal mucosa of IBD patients may promote chronic inflammation [45]. One study showed that dextran sodium sulfate (DSS)-induced colitis was ameliorated by pretreatment with the potent AhR agonist, TCDD, in mice [35]. A similar effect of TCDD was observed in a TNBS-induced murine model of colitis. Increased Foxp3 Treg populations in gut immune tissue were also detected in a mouse model [44]. Another *in vivo* study found that Beta-naphthoflavone (beta NF), a non-toxic agonist of AhR, suppressed the pathogenesis of the DSS-induced colitis through the inhibition of pro-inflammatory cytokine production, such as TNF-a and IL6. At the same time the author also found that DSS-evoked colitis was more severe in AhR knockout mice than in C57BL/6J wild type mice [46]. Six-formyl-indolo(3,2-b)carbazole (FICZ), generated from tryptophan by ultraviolet irradiation, has a higher AhR binding afflnity than TCDD and activates AhR inducing CYP1A1 mRNA expression. Monteleone’s group found that FICZ treatment demonstrated a protective effect against gastrointestinal tract inflammation both *in vivo* and *in vitro* [47]. TCDD treatment of intestinal lamina propria mononuclear cells *in vitro* reduced the level of cytokine IFN-r, while it increased the cytokine IL22 *in vitro*. *In vivo* study in mice found that FICZ treatment reduced TNBS, DSS and T cell transfer-induced colitis. The potential mechanism was thought to be due to down-regulation of the pro-inflammation cytokines including IFN-r, IL17 and TNF-a, and up-regulation of IL-22 production. This *in vivo* study also found that both neutralizing the IL-22 via injections of a blocking antibody of IL-22 and treatment with the AhR antagonist, 2-metal-2h-pyrazole-3-carboxylic acid, contributed to suppression of the production of cytokine IL-22 and acceleration of the symptoms of colitis. A recent study [37] showed a different molecular mechanism of the AhR signaling pathway in the process of improving the DSS-induced colitis in mouse model. The study showed that the AhR agonist, TCDD, caused partial demethylation of the Foxp3 promoter and hence enhanced Foxp3 expression, while it caused methylation of the IL-17 promoter and decreased the expression of IL-17, a pro-inflammatory cytokine secreted by Th17 cells.

### 3.2. Systemic Lupus Erythematosus

Several reports suggest that the prevalence of systemic lupus erythematosus (SLE), is also increasing in recent years. Environmental factors likely play a major role in such high morbidity [32]. The AhR signaling pathway plays important role both in the process of environmental exogenous chemicals metabolism and in immune system modulation. It is rational to speculate that AhR signaling also might be involved in SLE genesis and prognosis. Traditionally, SLE is identified as a Th2-type autoimmune disease, however, both Th17 and Treg cells are involved in the pathogenesis of SLE as the dynamic balance between these cells is damaged in SLE patients. Restoring the immune balance among Th1, Th17 and Treg cells, rather than exclusively focusing on Th17 cells, will yield better results for the treatment of SLE [48]. Furthermore, there is evidence that Th17 and Treg cells arise in a reciprocal manner, depending on potentially pro- or anti-inflammatory cytokines (IL-6, TGF-b, IL-23) and activation of specific transcription factors including AhR [3,48]. By the aid of the New Zealand Black/New Zealand White F1 hybrid (B/W) mouse model of SLE, one study showed that TCDD chronic exposure significantly lowered thymic and splenic weights, decreased the percentage of CD4 T cells, and also demonstrated an immunosuppressive effect on the prognosis of SLE, while DDT exposure markedly increased the incidence of albuminuria, reduced uterine weight and appeared to accelerate the development of albuminuria in lupus-prone mice, but had no measurable effects on immunity or mortality in that study [32].

### 3.3. Atopic Dermatitis-Related Inflammation

Generally, cytokines secreted by CD4 T cells stimulate keratinocytes to proliferate and recruit inflammatory cells into the skin, promoting epidermal hyperplasia and inflammation. Atopic dermatitis-related inflammation is a T helper 2 (Th2) and IL-22-producing CD4 (Th22) lymphocyte-mediated skin disease associated with loss-of-function mutations in the skin barrier gene, filaggrin (FLG) [49,50]. A recent study found that coal tar activated theAHR, resulting in induction of epidermal differentiation and contributed to restored expression of major skin barrier proteins, including filaggrin, which counteracted Th2 cytokine-mediated down-regulation of these proteins. They also found that the potential molecular mechanism was that coal tar treatment interfered with Th2 cytokine signaling via the dephosphorylation of Stat6, which was most likely due to AhR-mediated activation of the nuclear factor erythroid 2-related factor, an essential transcription factor that regulates expression of several antioxidant genes via binding to the antioxidant response element (ARE) and plays a crucial role in the cellular defence against oxidative stress [50]. In another study [51], TCDD exposure markedly increased the production of Th1 type cytokines (IFN-r), but not the Th2 cytokine (IL4), using spleen cells stimulated *in vitro* with a mitogen. TCDD exposure *in vivo* markedly increased mast cell infiltration and degranulation in picryl chloride (PC)-sensitized NC/Nga mice, which also suggested that TCDD exposure exacerbated atopic dermatitis.

### 3.4. Allergic Asthma and Lung Inflammation

Allergic asthma and lung inflammation is another kind of inflammatory autoimmune disease of the respiratory system. Millions of individuals worldwide are afflicted with acute and chronic respiratory diseases, which often occur as a result of altered immune responses, causing temporary and permanent disabilities and even death [52]. Pulmonary immunity is a multifaceted process, involving tolerance to self-antigens and innocuous environmental contaminants while at the same time preserving the capability to appropriately respond to invading pathogens. The balance between pro- and anti-inflammatory cells and their associated soluble cytokines directly influences outcome of the diseases. Investigators have demonstrated that the AhR signaling pathway plays an important role in modulating the immune response in various respiratory diseases and have shown that the respiratory system is sensitive to alterations in AhR expression or function, which also suggested the potential therapeutic effect of AhR ligands [52]. Asthma is characterized by the inflltration of the airways with various types of immune cells including eosinophils, mast cells, and T lymphocytes, leading to airway obstruction and airway remodeling, however, it is mainly caused by Th2 lymphocytes secreting IL-4 and IL-5 [53]. AhR specific agonists TCDD, Curcumin and FICZ have been shown to suppress allergic immune responses in several mice models, providing evidence for the involvement of AhR ligands in the pathogenesis of allergic asthma. An *in vitro* study found that treatment with FICZ decreased Th2 cell differentiation via the inhibition of expression of Th2 transcription factors, GATA3 and STAT6. An *in vivo* study of the ovalbumin-induced allergic asthma mouse model also found that FICZ treatment alleviated the pathogenesis of asthma and significantly reduced pulmonary eosinophilia, differentiation of Th2 cells and production of cytokines [54]. Certain natural-derived compounds could have this potential since they can function as chemo-preventers. These include flavonoids, which have been categorized into several groups. Flavonoids are natural plant pigments and their presence in many fruits and vegetables makes them available in the daily diet [55]. Quercetin (a plant-derived flavonoid with AhR agonistic activity) reduced IL-4 production and expression of Th2 transcription factor (GATA3), in lung tissues of ovalbumin (OVA)-sensitized and -challenged mice while increasing IFN-γ production and expression of Th1 transcription factor (T-bet) [56]. Curcumin (another AhR ligand, and widely used Indian spice) inhibited recruitment of eosinophils into the lung airways, airway hyper responsiveness, and expression of IL-4, in OVA-sensitized mice. OVA challenge resulted in activation of the production of inducible nitric oxide (iNOS) in lung tissue, inflammatory cytokines, recruitment of eosinophils to lung airways, and airway hyper-responsiveness to inhaled meth-choline, while pretreatment of mice with curcumin inhibited the OVA-induced inflammation. An *in vitro* study of an human airway epithelial cell line, A549, showed that curcumin decreased iNOS and nitric oxide (NO) production induced by IFN-gamma. Together, the results suggested that the AhR ligand curcumin contributed to anti-inflammatory activity in the murine asthma model and lung epithelial cell A549 through suppression of NO [57].

### 3.5. Rheumatoid Arthritis

Rheumatoid arthritis (RA) is the most common type of inflammatory and autoimmune arthritis, and is characterized by chronic inflammation of the synovium and subsequent joint destruction, while the pathogenic mechanisms of RA remain largely unknown. However, recent studies have demonstrated that many T cell subsets, including IL-17-producing T helper (Th17) and regulatory T (Treg) cells, play important roles in pathogenesis of RA [58]. On one hand, Th17 cells induced by TGF-β plus IL-6 or IL-21, secrete IL-17 isoforms that in association with other cytokines, such as IL-1 or TNF-α, synergistically activate synovial fibroblasts to produce matrix metalloproteinases. On the other hand, Treg cells, induced by TGF-β signaling, exert suppressive functions that are important for immunologic tolerance. The Th17/Treg cell balance is a new paradigm for autoimmunity [58]. It has been shown that the balance of Th17 and Treg cells contributes to the pathogenesis of several autoimmune diseases induced in animal models, including experimental autoimmune encephalomyelitis (EAE) and collagen-induced arthritis (CIA) [58,59]. At the same time, accumulating data from recent studies have shown that aryl hydrocarbon receptor (Ahr), plays a key role in regulating the differentiation of T cell subsets, especially Th17 and Treg cells, and consequently affects the development of several autoimmune diseases including RA [60,61]. Thus AhR targeted therapy is also potentially useful for rheumatoid arthritis. A previous study indicated that the development of experimental autoimmune arthritis depends on the presence of AhR in T cells [62]. AhR deficiency blocks cartilage destruction and reduces matrix metalloproteinase-3 levels and ameliorated collagen II-induced arthritis (CIA), a mouse model of rheumatoid arthritis (RA), while demonstrating that the pro-inflammatory effects of AhR in mice with collagen-induced arthritis can be mediated by Th17 cells and IL-22. Another study showed that TCDD-activated AhR signaling contributed to up-regulation of the expression of IL-1 beta, IL-6 and IL-8, that was transmitted via the NF-kappa B and ERK signaling cascades [63]. The study also found that AhR expression could be enhanced by the cytokine TNF-α, and cigarette smoking and exposure to TCDD contributed to promote RA inflammatory processes [63].

One previous study shows that resveratrol has adequate potency and nontoxicity to warrant clinical testing as a prophylactic agent against aryl hydrocarbon-induced pathology. Resveratrol inhibits the transactivation of several dioxin-inducible genes including cytochrome P-450 1A1 and interleukin-1 beta, both *in vitro* and *in vivo* [64]. Another research group found that resveratrol prevents the production of pro-inflammatory cytokines such as IL-6, TNF-α, IL-17, and IFN-γ in CIA mice, and also inhibits Th17 cell differentiation and autoantibody production by B cells, thus suppressing disease development [65].

### 3.6. Multiple Sclerosis

Strong auto-reactive Th17 activity and weak Tregs activity contribute significantly to development of multiple sclerosis (MS), an autoimmune inflammatory disease of the CNS that afflicts at least 400,000 people in the United States and 1.4 million people worldwide [66]. Experimental autoimmune encephalomyelitis (EAE), the best-characterized murine model of MS, resembles MS in many aspects. The important physiological role of ligand-activated transcription factor AHR as a regulator of T-reg and Th17 cell differentiation has been shown in mice, and studies on the potential therapeutic effects of AhR ligands have been increasingly investigated in the past few years [6]. Rouse *et al.* investigated the therapeutic efficacy of two AhR agonists, indole-3-carbinol (I3C) and diindolylmethane (DIM) against EAE. Their results demonstrated that pretreatment of EAE mice with I3C or DIM completely prevented clinical symptoms and cellular infiltration into the CNS. Also, post-treatment of EAE with I3C or DIM proved highly effective in curtailing the overall severity of the disease. The molecular mechanism involves AhR regulation of foxp3 induction and suppression of Th17 cells [59]. Interestingly, ligand-specific AhR functional diversity was also demonstrated [3,6]. Quintana *et al.* [6], showed that TCDD-activated AhR signaling induced differentiation of functional Treg cells, while inhibiting the differentiation of Th17 cell, and together this suppressed experimental autoimmune encephalomyelitis in the mouse model. Contrary to these results, FICZ-activated AhR signaling increased the differentiation of Th17 cell and interfered with Treg cell development, which together aggravated the development of EAE in mice. The different functions of two agonists of AhR, might be attributed to their different metabolic characteristics. FICZ has higher affinity even than TCDD but can be rapidly metabolized by AhR-induced P450 enzymes. TCDD also has high affinity, however, it cannot be metabolized rapidly *in vivo*. Similar works from Veldhoen *et al.* demonstrated that FICZ promoted the pathogenesis and progression of EAE in a mouse model in an AhR dependent manner [3]. Their results showed that FICZ treatment contributed to elevate Th17 cell differentiation and the production of inflammatory cytokine IL-22 both *in vitro* and *in vitro*. However, they did not completely support the opinion, of Quintana *et al.* that the differentiation and development of Treg cell might be interfered with by the FICZ-activated AhR pathway. The current understanding cannot explain the somewhat different outcomes. But notably, although most of the studies on the effect of AhR stimulation on the immune system have so far focused exclusively on TCDD, no direct measurements of AhR expression on highly purified polarized subsets of CD4 T cells have been reported. On the other hand, it is not yet fully understood whether the functional differences among different AhR ligands, in respect to their effect on immune system and immune response, rely more on effects on effector or regulatory T cells.

A recent study showed the potential therapeutic effect of another endogenous AhR agonist, 2-(1'*H*-indole-3'-carbonyl)-thiazole-4-carboxylic acid methyl ester (ITE) [10]. ITE-activated AhR signaling not only induced Treg differentiation and suppressed EAE in mice, but also induced tolerogenic dendritic cells that support Treg differentiation in a retinoic acid dependent manner, thereby promoting immunologic tolerance in the EAE mouse model. Resveratrol (3,5,4'-trihydroxystilbene), a phytoalexin derived from a variety of plants, is a competitive inhibitor of AhR (AhR antagonist). The potential therapeutic effect of resveratrol has also been investigated in past years using an animal model of multiple sclerosis. Singh *et al.* [67] found that resveratrol treatment ameliorated the clinical symptoms and inflammatory responses in an EAE mouse model, which also demonstrated the potential use of AhR antagonists as drug candidates for AhR-targeted therapy in the treatment of inflammatory and autoimmune diseases. A more recent report demonstrated that use of nanoparticles to co-administer ITE and a peptide containing the encephalitogenic epitope MOG35-55, which interfered with the differentiation of Th1 and Th17 cells and promoted the differentiation of Foxp3 Treg cells, eventually suppressed the development of EAE in mice [68].

Chemoprotective phytochemicals exhibit multiple activities and interact with several cellular receptors, including the AhR. Previous works have suggested that dietary phytochemicals exhibit AhR agonist as well as antagonist activities, although they might be within a cell context-dependent manner [69]. Recently, some phytochemicals as exogenous AhR ligands, have also been investigated in certain inflammatory diseases. Curcumin (also known as turmeric), a lipophilic polyphenol found in the rhizome of the plant Curcuma longa or herbal powder is also an exogenous AhR agonist. A wide range of studies has investigated the use of curcumin as an anti-inflammatory agent in both *in vitro* and *in vivo* systems. In clinical trials, the use of curcumin has been studied in various inflammatory conditions like ulcerative colitis, eye inflammation, pancreatic inflammation, joint inflammation, and a vast collection of other inflammatory conditions. The potential mechanism might be the down-regulation NF-kB, accompanied by the inhibition of COX-2 activation, inhibition lipoxygenases, and the pro-inflammatory cytokines, TNF-a, IL-1b, and IL-8 that are produced by a number of immune cell types [70]. Another potential exogenous AhR agonist, Baicalin, is a compound isolated from a Chinese herb. A previous study showed that Baicalin inhibited Th17 cell differentiation both *in vitro* and *in vivo*. Baicalin not only inhibited newly generated Th17 cells via up-regulating Foxp3 expression to suppress RORγt-mediated IL-17 expression in established Th17 cells *in vitro*, but also inhibited Th17 cell differentiation, restrained Th17 cells infiltration into kidney, and protected MRL/lpr mice against nephritis in the lupus-prone MRL/lpr mice (a inflammatory disorder mouse model) [71].

## 4. Summary and Outlook

The immune system in higher organisms is under integrated control and has the capacity to rapidly respond to environmental stimulators. The CD4 T helper cells play important roles not only in natural immunity but also in adaptive immunity. Since the CD4 T cells are central in the origin and regulation of autoimmunity, emphasis has been placed on the characterization of Th subsets and their possible roles in the inflammatory autoimmune process. The process of Th cell subset differentiation is tightly regulated to evoke appropriate immune response. Any disturbance of this process may result in the induction of undesirable inflammatory autoimmune diseases. The effector CD4 T cells subsets are associated with the pathogenesis of several autoimmune and inflammatory diseases, such as rheumatoid arthritis, systemic lupus erythematosus, multiple sclerosis, atopic dermatitis-related inflammation, inflammatory bowel disease, allergy and asthma, *etc.* Traditionally, the activation of AhR is well known for its ability to regulate the biochemical and toxic effects of environmental chemicals, however, increasingly exciting discoveries have been made indicating that the AhR signaling pathway can also regulate T-cell differentiation, specifically through activation of Foxp3^+^ regulatory T cells (Tregs) and down-regulation of the pro-inflammatory Th17 cells. Additionally, the importance of the AhR in immunological processes is illustrated by expression of this receptor in a majority of immune cell types. The AhR signaling pathway has been reported to influence a number of genes responsible for mediating inflammation and other immune responses. Together, such findings have opened new avenues of research on the possibility of targeting the AhR to treat inflammatory autoimmune diseases.

Although the AhR gene has been revealed to be highly conserved throughout evolution, it is important to note that the three dimensional structure of the AhR ligand binding domain has not been elucidated, and therefore the mechanism underlying ligand diversity and receptor conformational changes following binding remain unknown. The fact that the AhR gene has been so highly conserved, provides evidence of the fundamental importance of AhR in biological systems, not only *in vitro* but also *in vivo* [72,73]. AhR knockout (AhR^−/^^−^) animals suffer from a variety of organ disorders including a decline in the efficacy of their immune system [46]. On the other hand, ligand specific effects have been reported in the past few years [6,74], which point out that not only the AhR itself but also the particular ligand can induce a wide range of biological responses. Notably, there was another study showing that AhR expression is restricted to the Th17 cell subset and its activation results in the production of the Th17 cytokine, interleukin (IL)-22, in the CD4 T cell lineage both in mice and in human PMBCs [3]. FICZ-activated AhR signaling during the course of Th17 cell development markedly increased the proportion of Th17 T cells and their production of cytokines in an *in vitro* test system. *In vivo* experiments showed that FICZ treatment during induction of experimental autoimmune encephalomyelitis causes accelerated onset and increased pathology in wild-type mice, but not AhR-deficient mice. Such results suggest that AhR ligands may also represent co-factors in the development of autoimmune diseases in some specific conditions.

Interest in the AhR signaling path from a pharmacological perspective has been rapidly increasing and AhR has been revealed as a modulator of many diseases process. The role of AhR ligands in modulating CD4 T cell subset differentiation, transforming and cytokine production that contributes to promoting or inhibiting the process of inflammatory autoimmune disease, has been investigated both *in vitro* and *in vivo* in the past few years. Although the mechanisms of action have not been fully identified, AhR is known to bind to and be activated by a variety of compounds ranging from environmental contaminants to natural phyto-flavanoids or synthesized chemicals. Accumulating evidence demonstrates that AhR has a number of diverse ligands, both endogenous and exogenous. AhR ligands can vary greatly in their chemical structure and binding affinities. Those AhR ligands that possess a greater affinity for the receptor are often deemed more toxic. However, researchers have been searching for additional, less toxic AhR ligands that possess immune-modulatory and anti-cancer properties. As interest in the AhR and its ligands increases, it seems prudent to continue current research on the contributions of these ligands to immune regulation during the course of inflammatory diseases.

Given the role of AhR in mediating signals from environmental contaminants, it is not surprising that the first target genes identified for this pathway includes phase I and II xenobiotic metabolizing enzymes, However, data now indicates that the AhR pathway controls the expression of a variety of genes unrelated to xenobiotic metabolism, including genes encoding proteins involved in growth control, cytokines and other kinds of nuclear transcription factors. Thus the investigation of interactions of the AhR signaling pathway with other cell signaling pathways should be continued. Most recently, researchers have found that activation of AhR is required for the maintenance of lymphocytes as well as lymphoid architecture [25,75]. Many AhR-mediated immune regulatory mechanisms have been discovered, which will likely not only enhance our understanding of the molecular pathogenesis of autoimmune inflammation diseases, but also contribute to research and development of new potential drug candidates that target AhR or downstream elements of its signaling pathway for the therapy of inflammatory autoimmune diseases.

To develop such therapies, we need to establish a firm understanding of the mechanisms of action that might be involved during the course of AhR-mediated immune regulation. Essentially, the modulation models and effects exerted by ligand-induced AhR activation on the immune system are determined by a number of varying factors, which include the cell types involved, their activation status, type of ligand (agonist or antagonist), and the cytokines in the local microenvironment.

Furthermore, since each AhR ligand may cause different effects on diseases, elucidating the mechanisms of action by which specific AhR ligands regulate immune responses will provide a foundation for therapeutically targeting the molecule in a range of autoimmune and inflammatory diseases. Thus future experiments also need to be designed to determine the detailed molecular mechanisms by which each endogenous or exogenous AhR ligand triggers signaling, and the potential interaction with other cell signaling pathways.
